# AI in Personalized Learning for Older Adults and Intergenerational Engagement: A Systematic Review

**DOI:** 10.1192/j.eurpsy.2025.1760

**Published:** 2025-08-26

**Authors:** E. Tsiloni, E. Dragioti, M. Gouva, S. P. Vassilopoulos, M. Mentis

**Affiliations:** 1 Department of Educational Sciences and Social Work, University of Patras, Patra; 2 Department of Nursing, University of Ioannina, Ioannina, Greece

## Abstract

**Introduction:**

Artificial Intelligence (AI) is increasingly recognized as a powerful tool for customizing learning experiences, fundamentally transforming traditional teaching methods.

**Objectives:**

This systematic review aims to evaluate and synthesize current research on AI learning tools and their role in enhancing educational experiences for older adults, as well as in promoting intergenerational learning and engagement.

**Methods:**

Following PRISMA guidelines, a comprehensive review of both quantitative and qualitative data was conducted. Electronic databases such as PubMed, Scopus, and ERIC were searched up to October 20, 2024. The reference lists of included studies and relevant review articles were also thoroughly examined. The quality of the eligible studies was assessed using the Mixed Methods Appraisal Tool (MMAT).

**Results:**

Nine studies met the inclusion criteria. Findings indicate that personalized education for older adults can be delivered through various approaches, including interactive, user-friendly learning environments, training via virtual conversation agents and digital assistants, and automatic adjustments of pace and content to meet user needs (Figure 1). These approaches also facilitate and promote intergenerational learning (Figure 2).

**Image:**

**Figure:**
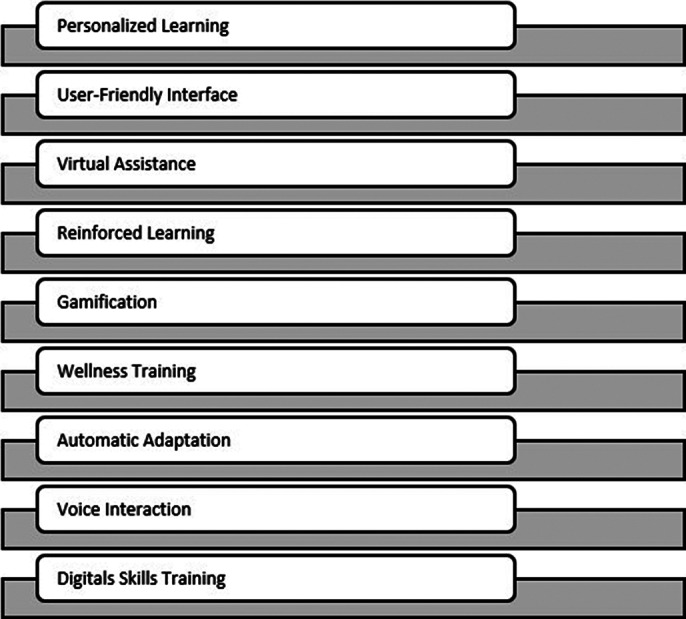

**Image 2:**

**Figure:**
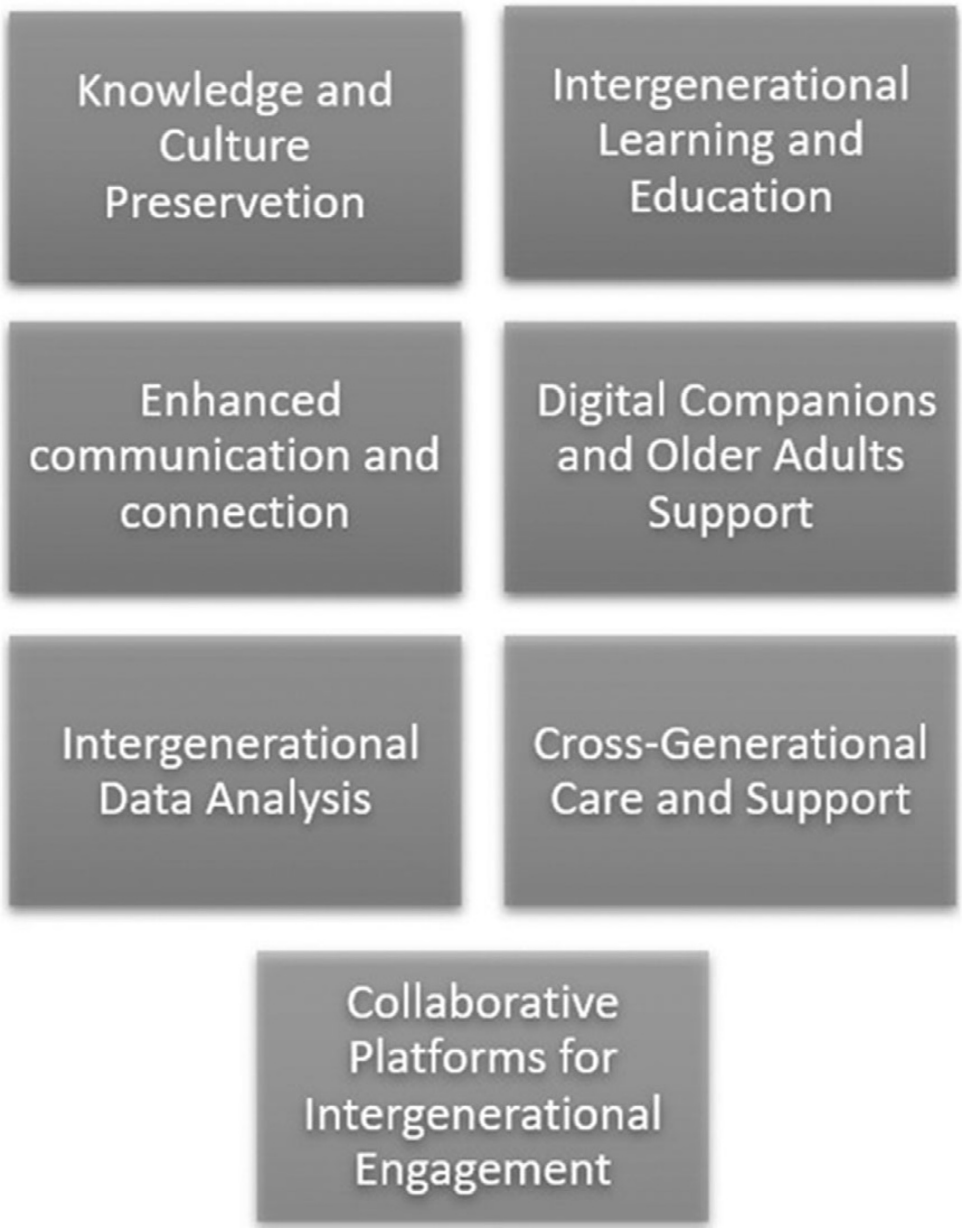

**Conclusions:**

Personalized education through artificial intelligence can significantly enhance older adults’ quality of life by promoting autonomy, expanding knowledge, supporting psychosocial well-being, and fostering intergenerational connections.

**Disclosure of Interest:**

None Declared

